# Nutrient and Dietary Patterns in Relation to the Pathogenesis of Postmenopausal Osteoporosis—A Literature Review

**DOI:** 10.3390/life10100220

**Published:** 2020-09-25

**Authors:** Bolaji Lilian Ilesanmi-Oyelere, Marlena C. Kruger

**Affiliations:** 1 School of Health Sciences, College of Health, Massey University, Tennent Drive, Palmerston North 4442, New Zealand; m.c.kruger@massey.ac.nz; 2 Riddet Institute, Massey University, Palmerston North 4442, New Zealand

**Keywords:** nutrient patterns, dietary patterns, postmenopausal osteoporosis, bone health, postmenopausal women

## Abstract

Postmenopausal women tend to be susceptible to primary osteoporosis due to its association with oestrogen deficiency. There is emerging evidence that an unhealthy dietary pattern drives an increase in the risk of postmenopausal osteoporosis (PO), whereas a healthy dietary pattern may decrease its occurrence. In this narrative literature review, we sought to review the role of nutrient and dietary patterns in the pathogenesis of PO. Therefore, we searched and reported all research articles from 2001 to May 2020 in Web of Science, Cinahl and Scopus that have researched a relationship between nutrient and/or dietary patterns and postmenopausal osteoporosis. Nutrients such as calcium, phosphorus, magnesium and vitamin D have been proven to be beneficial for bone health. Meanwhile, for the dietary patterns, foods such as dairy products especially milk, fibre and protein-rich foods, e.g., meat were directly linked to a positive association with bone mineral density (BMD). Likewise, fruits, vegetables and probiotic and prebiotic foods were reported for its positive relationship with BMD. Therefore, aside from physical activity, nutrition and diet in adequate proportions are suggested to be an important tool for ameliorating osteoporosis and bone health issues in older age.

## 1. Introduction

Osteoporosis is a major public health concern with the ageing populations [1]. Worldwide, 8.9 million fractures occur annually which results in an osteoporotic fracture every 3 s [2].

Postmenopausal osteoporosis is a condition on the rise amongst aged women as the world demography experiences marked ageing of the population. Globally, New Zealand is amongst one of the most affected by the burden of the disease. Postmenopausal osteoporosis is characterized by increased low-grade inflammation contributing to low bone mass and degradation of bone mineral content resulting in bone loss and/or fractures [1,2].

Although the pathogenesis of osteoporosis is multifactorial, key drivers include oestrogen deficiency, poor dietary habits, chronic inflammation, smoking, excessive alcohol consumption and sedentary lifestyle. However, diet regulates the composition and function of the human gut microbiota with recent evidence suggesting that the gut microbiome plays essential roles in the host energy homeostasis, immune system enablement and metabolic function and health [3].

Menopausal hormone replacement therapy (HRT) has been employed in the treatment of menopausal treatments. However, its risks have also been documented which include increased occurrence of breast cancer, stroke, venous thromboembolism (VTE) and risk of coronary artery disease but the “timing hypothesis” has been suggested for a possible amelioration if administered early in menopause [4].

Nutraceuticals, also known as alternative pharmaceuticals products made from plants and foods which have medicinal properties, are non-hormonal natural therapies or approach to menopausal symptoms. These include phytoestrogenic plants or isoflavones, antioxidants, dietary supplements and fortified dairy products [5].

Nutrition and lifestyle changes are essential in promoting health and in the prevention of metabolic diseases such as osteoporosis. Many nutrients are known to interact with each other thereby influencing their bioavailability and absorption [3]. Several key nutrients are known to affect bone mineral content (BMC) and bone mineral density (BMD). These nutrients, however, occur together in foods and dietary patterns, therefore the need to study the diet in its entirety. Unhealthy dietary patterns are known to be associated with some chronic diseases such as diabetes and cardiovascular disease [4]. Likewise, nutrients such as calcium and vitamin D are well-established as nutritional drivers in the maintenance of normal bone metabolism. Additionally, nutrients such as potassium, zinc, magnesium, iron, copper, vitamin C and vitamin K are micronutrients rich in fruits and vegetables that are beneficial for bone metabolism. However, the overall effects of dietary choices on bone health are not well understood and therefore need further research and discussion.

There are two main holistic methodologies used in describing and quantifying nutrient and/or dietary patterns/habits: 1. The a posteriori (data-driven) dietary pattern approach, i.e., the use of statistical methods such as principal component analysis (PCA) or factor analysis, reduced rank regression (RRR), cluster analysis and partial least square to generate dietary patterns from data collected; and 2. The a priori dietary pattern approach, i.e., the use of created or predefined dietary indexes on the basis of existing knowledge in nutrition usually complying with dietary guidelines and recommendations [5].

The aim of this review was to investigate and discuss the reported relationships observed between nutrient and dietary patterns and bone health status (BMD, bone biomarkers and fracture risks) in postmenopausal women.

## 2. Current Evidence and Status of Knowledge

### 2.1. The Relationship between Nutrient Patterns and Postmenopausal Osteoporosis

Research shows there is a relation between nutrient patterns and postmenopausal osteoporosis; however, results from studies are heterogeneous and therefore no conclusive nutrient pattern has been proposed. To date, only two studies have explored the relationship between nutrient patterns and bone health exclusively in postmenopausal women.

The first study by Karamati et al., 2014 indicated that a nutrient pattern (NP1) high in folate, fiber, vitamin B6, potassium, vitamin A, vitamin C, β-carotene, vitamin K, magnesium, copper and manganese was positively associated with lumbar spine BMD. These nutrients are particularly rich in fruits and vegetables. These antioxidant micronutrients are important for the formation and maintenance of bone cells and the structure required for normal bone metabolism. However, they failed to find any correlation with the well-known nutrients that are important for bone health in their NP2 which was high in vitamin B2, protein, calcium, phosphorus, zinc, vitamin B12, vitamin D and low in vitamin E. The explanation was that although protein intake levels have been associated with bone health benefits, the influence of protein intakes generally depends on a balanced whole diet in terms of acid-producing potential. Acid/base balance is important to avoid urinary calcium loss with acid-forming foods such as processed meat, shellfish and pastries. The NP3 with high intakes of dietary fats and low intakes of carbohydrate, vitamin B1 and fiber was likewise not correlated with BMD [6].

The second study by Ilesanmi-Oyelere et al., 2019 found a positive association between NP1 (characterised by high riboflavin, phosphorus, calcium, sugars, potassium, vitamin B6, carbohydrate and magnesium) and lumbar spine, femoral neck and whole-body BMD. These nutrients are particularly rich in eggs, lean meats, milk, milk products and some fruits and vegetables. NP2 (high in dietary fats, vitamin E, alpha and beta carotene) was negatively associated with BMD while NP3 (also characterised by high fat, protein, zinc and cholesterol with low intakes of alpha and beta carotene and vitamin C) was not associated with BMD at all sites (Table 1).

### 2.2. Dietary Pattern Analyses and Bone Health in Postmenopausal Osteoporosis

Generating dietary patterns with correlated foods are important to investigate diet due to the complexity and interaction of various nutrients and foods. Studies have used dietary patterns generated from foods to give a view of the association between dietary intakes and BMD/BMC, bone biomarkers, osteoporosis and fractures (Table 2). 

Traditional Western-style diets that are characterised by processed foods high in salt, fats and sugar have been researched and positively associated with osteoporosis as is evidenced in six studies that explored dietary patterns and bone health status [10,11,12,13,14]. Similarly, energy-dense foods such as white rice, wheat and grains have been associated with the risk of fractures during postmenopause [15]. These patterns of foods have therefore been labelled “unhealthy” and are known to drive the risk of many metabolic diseases including osteoporosis and consequently fractures. 

On the other hand, foods such as milk, low-fat dairy, fruit, vegetables and nutrient-dense foods have been associated with high BMD and lower risk of osteoporosis or fractures. These food patterns have been termed “healthy” and/or “prudent” dietary patterns.

### 2.3. Dietary Patterns Score/index and Bone Health in Postmenopausal Osteoporosis

Some studies have used the dietary pattern score/index in association with BMD/BMC, bone biomarkers and fractures in postmenopausal women as is shown in Table 3. The Mediterranean score indicates compliance with the Mediterranean diet. A traditional Mediterranean diet is rich in the intake of vegetables, fruit, nuts and olive oil but low in saturated fats, moderately high intake of fish, low to moderate intake of dairy and lesser intake of meat and poultry as well as moderate intake of wine [22,23,24]. In general, the Mediterranean diet score/index was directly associated with BMD and inversely associated with fracture risk [22,23]. Meanwhile, the Healthy Eating Index (HEI) that measures the quality of diet and how well a particular set of foods aligns with the dietary guidelines for Americans has been reported as having no significant association for both the HEI 2005 and 2010 [25,26]. However, Zheng et al., 2014 reported an inverse association between HEI 2005 and hip fracture risk. Furthermore, a study by De Jonge et al., 2015 reported a direct association between the BMD Diet Score and the Healthy Diet Indicator with femoral neck BMD in a large number of postmenopausal women based in the Netherlands [24].

On the other hand, the Dietary Inflammatory Index (DII) that assesses the inflammatory potential of a diet was inversely associated with BMD as was shown by two separate studies from the United States of America and Iran [25,26], these indicating the relationship between inflammation and bone degeneration.

## 3. Discussion

In this review, we discussed various studies on the relationship between nutrient and dietary patterns and BMD/BMC, bone biomarkers and fractures in postmenopausal women. Studies on the nutrient patterns and BMD, although conducted in different communities and settings, indicate the importance of phosphorus, riboflavin, potassium, calcium, magnesium, vitamin B6, vitamin D, protein, fiber, vitamin K and folate from fruits and vegetables as well as milk intake [6,7,8]. 

Dietary patterns help elaborate the health attributes of food groups; in factor or cluster analysis as a combination of foods and in the reduced rank regression (RRR) analysis method to represent groups of food with health outcomes. It is not surprising that foods such as low-fat dairy, legumes, nuts, olive oil, fish, fruits and vegetables have emerged in this study as important for the prevention of non-communicable diseases such as osteoporosis and fractures. Similarly, Mediterranean-based dietary patterns have been reported to be directly associated with calcium absorption and therefore bone health status in men [31]. Although data-driven dietary patterns are known not to be reproducible and comparable across studies with the explanation that subjectivity of the approach or the fact that real habits of dietary intake across populations differ [5]. Data-driven dietary patterns, however, have the advantage of assessing the real dietary/food patterns in the populations.

On the other hand, processed and/or refined foods such as French fries, hamburgers, biscuits and cookies, and carbonated drinks known as the Western (unhealthy) food patterns, have frequently been inversely correlated with BMD and bone parameters both in children and aged adults. Sweet foods, coffee and tea have also been reported to be inversely correlated with BMD/BMC [18]. Similarly, foods high in sugar such as added sugar fruit drinks, chocolate, and confectionery and high-fat foods such as processed meat, French fries, mayonnaise and desserts [17] were all negatively correlated with BMD and/or BMC. Investigations on starchy-foods (rice) patterns have shown that a high intake of rice in foods was associated with a higher risk of osteoporosis [11,13]. Our nutrient and dietary-based investigation into the pathogenesis of osteoporosis in postmenopausal women showed the importance of an adequate and balanced diet.

Overall, the choice of foods is important for optimum health during aging and a “healthy” diet rich in vegetables and milk or a Mediterranean style diet may be beneficial for bone health in comparison to a Western-style traditional dietary pattern. The implementation of policies for an increase in the dietary intake of vegetables, fruits, non-refined grains and low-fat milk is warranted from childhood to adulthood.

## Figures and Tables

**Table 1:** Nutrient patterns and bone mineral density in postmenopausal women.

Study, Location and Design	Participants Information	Diet Assessment/Method	Nutrient Patterns Generated	Main Results	Ref.
Factor analysis BMD/BMC					
Postmenopausal Iranian women, Iran, cross-sectional	160 postmenopausal women, age 50–85 years	Validated 168-item food frequency questionnaire/LS BMD and FN BMD by DXA	NP1 was high in folate, total fiber, vitamin B_6_, potassium, vitamin A, C, K, β-carotene, magnesium, copper and manganese. NP2 was high in vitamin B_2_, protein, calcium, phosphorus, zinc, vitamin B_12_, and vitamin D and low in vitamin E. NP3 was high in total fat, monounsaturated fatty acids, saturated fatty acids and polyunsaturated fatty acids with low levels of carbohydrate and vitamin B_1_.	NP1 which was associated with high intakes of fruits and vegetables and low intakes of cereal was significantly positively correlated with lumbar spine BMD but not the femoral neck. NP2 and NP3 were not significantly associated with BMD at any of the sites.	Karamati et al., 2014 [6]
North West Adelaide Health Study, Australia, cross-sectional	1135 adults, median age 62 years	Validated food frequency/BMD by DXA	Mixed-source pattern was high in phosphorus, niacin, starch/dextrins and riboflavin. Animal-sourced pattern high in palmitoleic acid, cholesterol and omega-6. Plant-sourced pattern high in β-carotene, lutein and zeaxanthin and vitamin C.	Mixed-source nutrient pattern was positively associated with BMD. No independent and statistically significant associations between animal- and plant-sourced nutrient patterns and BMD were found	Melaku et al., 2017 [7]
“Bug‘n’Bones” study, New Zealand, cross-sectional	101 postmenopausal women, age 54–81 years	3-day diet diary/LS, FN and hip BMD by DXA	NP1 high in riboflavin, phosphorus, calcium, sugars, potassium, vitamin B6, carbohydrate and magnesium and NP2 high in dietary fats and fatty acids, vitamin E and NP3 high in fats, protein, cholesterol and low levels of vitamin C, α- and β-carotene.	NP1 was positively correlated with the spine, hip and femoral neck BMD while NP2 was negatively correlated with hip and whole-body BMD.	Ilesanmi-Oyelere et al., 2019 [8]
**Fractures**					
Bordeaux sample of the Three-City Study, France, longitudinal	934 women and 548 men, aged 68–95 y	24-h dietary recal and a food frequency questionnaire/Hip, wrist, and vertebrae fracture; self-reported incidence	(1) Nutrient-dense; high in calcium, phosphorus, iron, B vitamins, vitamin C and E, protein and unsaturated fats. (2) retinol, vitamin B-12, folate, iron; (3) southwestern French high in proteins, fats, alcohol, calcium, phosphorus, vitamin D and B12	Pattern (1) was inversely associated with risk of wrist and overall fractures; pattern (3) was inversely associated with risk of hip fracture	Samieri et al., 2013 [9]

BMD = bone mineral density; BMC = bone mineral content; LS = lumbar spine; FN = femoral neck; DXA = dual energy X-ray absorptiometry; NP = nutrient pattern.

**Table 2:** Dietary patterns and bone health in postmenopausal women.

Study, Location and Design	Participants Information	Diet Assessment/Method	Dietary Patterns Generated	Main Results	Ref.
Factor analysis BMD/BMC					
Co-twin controlled study, United Kingdom, cross-sectional	4928 postmenopausal women, aged 56 ± 12 y	Validated 131-item food-frequency Questionnaire/FN BMD, total hip BMD, LS BMD by DXA	(1) Fruit and vegetables, (2) high intake of alcohol, (3) traditional English, (4) dieting, (5) low meat intake	Pattern (3) was inversely associated with FN BMD	Fairweather-Tait et al., 2011 [10]
Annual health check-up program, Japan, cross-sectional	293 postmenopausal women, aged 60 ± 6 y	Modified validated simple food frequency questionnaire (FFQ)/33% Radial BMD by DXA	(1) Carotene, (2) retinol, (3) β-cryptoxanthin	Pattern (2) was inversely associated with BMD and pattern (3) was positively associated with BMD	Sugiura et al., 2011 [11]
Postmenopausal Iranian women, Iran, cross-sectional	160 women, aged 50–85 y	Validated 168-item food frequency questionnaire/LS BMD and FN BMD by DXA	(1) Folate, total fiber, vitamin B-6, potassium, vitamins A, C, and K, β-carotene, magnesium, copper, and manganese; (2) vitamin B-2, protein, calcium, phosphorus, zinc, vitamin B-12, vitamin D, and low vitamin E; (3) total fat, MUFAs, SFAs, PUFAs, and low carbohydrate and vitamin B-1	Pattern (1) was directly associated with LS BMD	Karamati et al., 2012 [12]
2-y prospective study of postmenopausal women, China, cross-sectional	282, 212, and 202 women at baseline, year 1 and year 2, respectively, aged 50–65 y at baseline	Validated 80-item food frequency questionnaire/Hip BMD (FN, trochanter, and Ward’s) LS BMD, TB BMD by DXA	Pattern (1): rice, cooked wheat food, fried food and other grains, and fruits; pattern (2): milk and root vegetables	Pattern (1) was inversely associated with hip and LS BMD; pattern (2) was directly associated with hip BMD.	Chen et al., 2015 [13]
Brazilian postmenopausal women with osteoporosis, Brazil, cross-sectional	156 women, aged ≥ 45 y; mean age 68 ± 9 y	3-day food diary/LS BMD, total femur BMD, FN BMD, TB BMD by DXA	(1) Healthy; high in vegetables, fruit and fresh juices, and tubers (2) red meat and refined cereals; (3) low-fat dairy; (4) sweet foods, coffee, and tea; (5) Western; high in snacks, pizzas and pies, soft drinks and fats	Pattern (4) was inversely associated with total femur and TB BMD	de França et al., 2016 [14]
**Bone Biomarkers**					
Aberdeen Prospective Osteoporosis Screening Study, Scotland, cross-sectional	3236 women, aged 50–59 y	Validated 98-foods FFQ/Bone resorption biomarkers: urine fPYD: Cr and fDPD:Cr ratios; bone formation biomarker: serum P1NP	(1) Healthy foods with high intakes of fruit and vegetables (2) processed foods, (3) bread and butter, (4) fish and chips, (5) snack foods with high intakes of confectionery, crisps, nuts and sauces	Pattern (1) was inversely associated with bone resorption biomarkers	Hardcastle et al., 2011 [15]
Canadian Multicenter Osteoporosis Study, Canada, longitudinal	754 women, 318 men, aged 63 ± 11 y	Food frequency questionnaire/Bone resorption biomarkers: CTX; bone formation biomarker: BAP; PTH; blood samples collected in year 5 of study	(1) Prudent, high in vegetables, fruit, whole grains, and legumes and (2) Western, high in soft drinks, potato chips and French fries, processed meats, and desserts	Pattern (1) was inversely associated with CTX in women and PTH in men; pattern (2) was directly associated with BAP and CTX in women	Langsetmo et al., 2016 [16]
**Osteoporosis**					
Korean Health and Nutrition Examination Survey 2008–2010, Korea, cross-sectional	735 postmenopausal women, aged 64 ± 9 y	24-h recall/Osteoporosis by LS and femur (FN, trochanter, intertrochanter, Ward’s, and total) BMD T-score by DXA	(1) Meat, alcohol, and sugar; (2) vegetables and soy sauce; (3) white rice, kimchi, and seaweed; (4) dairy and fruit	Pattern (4) was inversely associated with risk of osteoporosis and pattern (3) was directly associated with risk of osteoporosis	Shin and Joung 2013 [17]
Korean Genome and Epidemiology Study, Korea, longitudinal	1464 postmenopausal women, 4-y follow-up	103-food item, semiquantitative food frequency questionnaire (SQFFQ)/Osteoporosis incidence by SOS T-score at the mid-radius and tibia shaft by ultrasound	(1) Traditional (high in rice, kimchi and vegetable intake, (2) dairy (high in dairy products, milk and green tea intake), (3) Western (high in fat, sugar and bread)	Pattern (2) was inversely associated with and patterns (1) and (3) were directly associated with risk of osteoporosis	Park et al., 2012 [18]
**Fractures**					
Canadian Multicenter Osteoporosis Study, Canada, longitudinal	3539 postmenopausal women, aged 67 ± 8 y and 1649 men, aged ≥50 y (64 ± 10 y)	Self-administered FFQ/Low-trauma fractures by year 10 of study by self-reported interviews	(1) Nutrient-dense (2) energy-dense (Western)	Pattern (1) was inversely associated with risk of fracture in men and women	Langsetmo et al., 2011 [19]
**Cluster Analysis**					
Framingham Osteoporosis Study, United States, cross-sectional	562 women and 345 men, aged 69–93 y	Validated FFQ/FN BMD, Ward’s area BMD, and trochanter BMD by Lunar dual photon absorptiometry; 33% radius shaft BMD by Lunar single-photon absorptiometry	(1) Meat, dairy, and bread; (2) meat and sweet baked products; (3) sweet baked products; (4) alcohol; (5) candy, (6) fruit, vegetables, and cereal	Cluster (6) was directly associated with FN BMD, Ward’s BMD, and trochanter BMD when compared with clusters 2–4 in men; cluster (5) was inversely associated with FN BMD, Ward’s BMD, and radius BMD when compared with cluster (6) in men cluster (5) was negatively associated with radius BMD when compared with clusters (1), (2), (4), and (6) in women.	Tucker et al., 2002 [20]
In CHIANTI Study, Italy, longitudinal	434 women, aged 65–94 y (75 ± 7 y)	236-foods European Prospective Investigation into Cancer and Nutrition (EPIC) questionnaire/Total and trabecular BMD at 4% and cortical BMD at 38% tibia by pQCT; BMD variation over 6 y	(1) Lower intake of energy (30 kcal/kg IBW) and bone-related nutrients; (2) higher intake of energy (44 kcal/kg IBW) and bone-related nutrients	Cluster (2) was directly associated with cortical BMD and inversely associated with cortical BMD loss over 6 y compared with cluster (1)	Pedone et al., 2011 [21]

BMD = bone mineral density; BMC = bone mineral content; LS = lumbar spine; FN = femoral neck; DXA = dual energy X-ray absorptiometry; FFQ = food frequency questionnaire; TB = total body; fPYD = free pyridinoline; fDPD = free deoxypyridinoline; Cr = creatinine; CTX = C-terminal telopeptide; BAP = bone alkaline phosphatase; PTH = parathyroid hormone; SOS = speed of sound; IBW = ideal body weight; pQCT = peripheral quantitative computed tomography.

**Table 3:** Dietary pattern score/index and bone health in postmenopausal women.

Study, Location and Design	Participants’ Information	Diet Assessment/Method	Dietary Patterns Score/Index Generated	Main Results	Ref.
BMD/BMC					
Southern Spain women study, Spain, cross-sectional	100 premenopausal (aged 34 ± 7 y), 100 postmenopausal (aged 54 ± 6 y) women, aged 18–65 y	Validated semi-quantitative FFQ/Calcaneus BMD by DXA	Mediterranean Diet Score (MDS)	MDS was directly associated with BMD in all subjects	Rivas et al., 2013 [23]
Postmenopausal women, Iran, cross-sectional	160 postmenopausal women, aged 50–85 y	Validated semi-quantitative FFQ/FN BMD and LS BMD by DXA	Dietary Inflammatory Index (DII)	DII inversely associated with LS BMD	Shivappa et al., 2016 [27]
The Rotterdam Study, Netherlands, longitudinal and cross-sectional	2932 women and 2211 men, aged ≥ 55 y at baseline (median: 67 y; IQR: 61–73 y)	170 food items semi-quantitative FFQ/FN BMD by DXA, at baseline and three subsequent visits	BMD Diet Score	Directly associated with FN BMD	De Jonge et al., 2015 [28]
			Healthy Diet Indicator	Directly associated with FN BMD, but three times weaker than BMD Diet Score	
The women’s Health Initiative, USA, observational study and clinical trial	160,191 women between 50–79 y	BMD of the total hip, lumbar spine (L2–L4), and total body Women’s Health Initiative (WHI) FFQ/Total hip, LS and total body BMD by DXA	Dietary Inflammatory Index (DII)	Less inflammatory dietary pattern was associated with less BMD loss in postmenopausal women.	Orchard et al., 2017 [29]
**Bone Biomarkers**					
NHANES 1999–2002, United States, cross-sectional	827 postmenopausal women aged ≥ 45 y	24-h dietary recall interview/Bone formation: serum BAP; bone resorption: urinary N-telopeptide or creatinine	Healthy Eating Index 2005	No association was found	Hamidi et al., 2011 [25]
**Osteoporosis**					
Fifth Korean National Health and Nutritional Examination Survey (2010), Korea, cross-sectional	847 postmenopausal women	24-h dietary recall/Osteoporosis and osteopenia based on WHO BMD T-score criteria	Mean Nutrient Adequac Ratio	No association was found	Go et al., 2014 [30]
			Dietary Diversity Score	Inversely associated with risk of osteoporosis and osteopenia	
			Calcium source assessmen	Milk, anchovy, and sea mustard were inversely associated with risk of osteoporosis and osteopenia	
			Food Group Intake Pattern	No association was found	
**Fractures**					
China, case–control	549 women pairs and 177 men pairs, age-matched; aged 55–80 y	Validated 79-item food-frequency questionnaire (FFQ)/Hip fracture	Healthy Eating Index 2005	Inversely associated with hip fracture risk	Zeng et al., 2014 [31]
Three-City Study, France, longitudinal	932 women and 550 men, aged ≥ 67 y at baseline, 8 y follow-up	FFQ and 24-h dietary recall/Hip, vertebral, and wrist fractures; self-reported every biennial interview	Mediterranean Diet Score	No significant association	Feart et al., 2013 [22]
Women’s Health Initiative observational study, United States, longitudinal	90,014 postmenopausal women, aged 50–79 y (63 ± 7) at baseline, 16–21 y follow-up	WHI FFQ/Total and hip fracture	Alternate Mediterranean Score	Alternate Mediterranean score inversely associated with hip fracture risk	Haring et al., 2016 [26]
			Healthy Eating Index 2010	No significant association	
			Alternative Healthy Eating Index 2010	No significant association	
			Dietary Approaches to Stop Hypertension	No significant association	

BMD = bone mineral density; BMC = bone mineral content; LS = lumbar spine; FN = femoral neck; DXA = dual energy X-ray absorptiometry; FFQ = food frequency questionnaire; MDS = Mediterranean diet score; DII = dietary inflammatory index; WHI = women’s health initiative; WHO = World Health Organisation.
